# No Evidence of Prolonged Hendra Virus Shedding by 2 Patients, Australia

**DOI:** 10.3201/eid1812.120722

**Published:** 2012-12

**Authors:** Carmel Taylor, Elliott G. Playford, William J.H. McBride, Jamie McMahon, David Warrilow

**Affiliations:** Queensland Health Forensic and Scientific Services, Archerfield, Queensland, Australia (C. Taylor, J. McMahon, D. Warrilow);; Princess Alexandra Hospital, Brisbane, Queensland, Australia (E.G. Playford);; University of Queensland School of Medicine, Brisbane (E.G. Playford);; and James Cook University School of Medicine and Dentistry, Cairns, Queensland, Australia (W.J.H. McBride)

**Keywords:** Hendra virus, viruses, PCR, virus shedding, outbreak, zoonoses, Australia

## Abstract

To better understand the natural history of Hendra virus infection and its tendency to relapse, 2 humans infected with this virus were monitored after acute infection. Virus was not detected in blood samples when patients were followed-up at 2 and 6 years. Thus, no evidence was found for prolonged virus shedding.

Most virus infections resolve after an acute phase. A small subset can cause persistent infection and result in continual shedding of virus, and others use a latency mechanism to evade the host immune response. Hendra and Nipah viruses (family *Paramyxoviridae*, genus *Henipavirus*) can cause respiratory disease and encephalitis in humans. Hendra virus infection is acquired by close contact with horses infected by spillover infection from fruit bats, which are the natural reservoir for these viruses (*1*).

In a small proportion of cases, relapse with encephalitis after a mild acute phase is a feature of henipavirus infection and is often fatal. For example, several cases of relapsing encephalitis caused by Nipah infection in humans have been reported (*2*–*4*), as well as 1 case caused by Hendra virus in a human (*5*,*6*). Whether virus shedding occurs after an acute infection of any severity is currently unclear. Because standard veterinary practice in Australia is to destroy animals that survive natural or experimental Hendra virus infection (*7*), long-term monitoring for virus shedding has not been possible.

To address the nature of viral persistence in cases of Hendra virus infection, virus shedding and serologic changes were monitored in 2 of 3 infected persons. In addition, multiple samples from 1 of these persons and from 2 persons who died were compared to determine the most appropriate specimen type for detection by quantitative reverse transcription PCR (qRT-PCR).

## The Study

Of 7 recognized humans infected with Hendra virus, 3 survived. Of these 3 persons, clinical samples were available for 2 survivors. Patient 1 was a 25-year-old woman (veterinarian) in whom a self-limited influenza-like illness (ILI) developed in November 2004 (*8*). Patient 2 was a 21-year-old woman (veterinary nurse) who showed development of an ILI that progressed to acute encephalitis in July 2008 (*9*). Although she survived, patient 2 showed persistent, postencephalitic, high-level cognitive deficits. Currently, both patients do not show clinical evidence of relapse.

For this study, specimens were obtained from both patients at various times during acute-phase infection and during the convalescent phase. Blood was tested for IgG and IgM against Hendra virus. Nasopharyngeal aspirate (NPA), blood, urine, and cerebrospinal fluid were tested for viral RNA by using qRT-PCR.

Both patients showed IgM and IgG responses to Hendra virus. For patient 1, no specimens were collected in the months after the acute phase and by 12 months, no IgM could be detected. However, IgG reactivity has been maintained for 6 years (Figure). Patient 2 has maintained IgM and IgG reactivity for >1.5 years after initial examination. The prolonged IgM response in patient 2 might have been caused by persistent infection or associated with more severe acute encephalitic manifestations. A prolonged IgM response has been observed in persons with West Nile virus meningitis, and encephalitis and may be a general aspect of central nervous system involvement (*12*,*13*).

**Figure:**
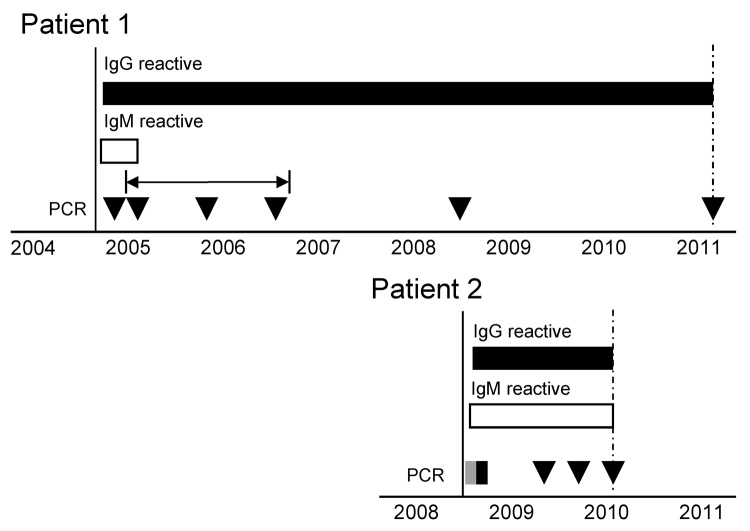
Serologic and quantitative reverse transcription PCR (qRT-PCR) results for samples from 2 patients infected with Hendra virus, Australia. Testing was performed from the time at which symptoms first developed (black vertical line) until the most recent sample indicated (dashed vertical arrows). IgG (black bars) and IgM (white bars) reactivity was determined by using a modified microsphere immunoassay (*10*), and a positive control serum sample to determine the cutoff value. Virus RNA was detected (gray box) or not detected (black box or triangles) by using a qRT-PCR (*11*). The horizontal arrow indicates 3 samples stored at −80°C and tested retrospectively for Hendra virus RNA. Positive and negative controls were included in all tests and showed expected results.

Viral RNA was not detected in any specimen from patient 1 during the acute phase or at the 6-year follow-up (Figure). Multiple samples from patient 2 were positive for virus during a 1-month period during the acute phase (Figure, Table). However, no samples showed positive results for Hendra virus RNA on subsequent follow-up. Thus, neither patient showed evidence of virus shedding after the acute phase (ILI or encephalitis).

To facilitate future PCR testing, we assessed various sample types for their suitability for detection of Hendra virus RNA during the acute phase of infection. In addition to patient 2, samples from 2 other case-patients with Hendra virus infection not described in this report (patients 3 and 4) were tested. Patient 1 was not included because virus shedding was not detected at any time during her infection. Times chosen were from the earliest to the latest time point at which a positive result was obtained by qRT-PCR (Table).

**Table:** Samples from patients infected with Hendra virus, Australia*

Sample type	No. positive/no. tested (%)		
	Patient 2†	Patient 3‡	Patient 4‡
Blood	7/18 (39)	6/11 (55)	22/22 (100)
Urine	9/17 (53)	7/7 (100)	19/22 (86)
NPA or swab specimen	10/13 (77)	3/11 (27)	19/ 20 (95)

*NPA, nasopharyngeal aspirate. †Human infected with Hendra virus; virus shed 3–6 weeks postexposure. ‡Two other humans infected with Hendra virus; virus was shed 3–5 weeks postexposure.

Hendra virus RNA was detected in blood, NPA (or swab specimen), and urine, confirming the suitability of these sample types. Frequency of detection varied for 3 patients. None of the 3 sample types was consistently better than another, and all were suitable choices for diagnostic purposes. Hendra virus RNA was also detected in cerebrospinal fluid, but there were too few samples to determine whether this material was a reliable sample type.

## Conclusions

Serologic and PCR results were reported for the 2 patients during the early phase of infection (*8*,*9*). Our study followed-up survivors of Hendra virus infection, and found no evidence for prolonged shedding of virus after acute infection. Because of the small total number of human cases and the high case-fatality rate, there are only 3 known surviving patients with Hendra virus infection, 2 whom were included in this study. Therefore, our results provide useful information for clinicians and public health officials for treating individual patients and minimizing the risk for transmission.

However, because of the small number of samples tested in this study, prolonged shedding after acute Hendra virus infection cannot be ruled out. In 1995, a patient who had an episode of aseptic meningitis after caring for 2 Hendra virus–infected horses had a relapse 13 months after the initial episode (*5*). This patient also showed the potential for virus shedding during management of disease survivors. Thus, further monitoring of current and future survivors and experiments using a suitable animal model is required to answer this question.

The mechanism by which henipaviruses remain dormant is unknown. Virus has not been isolated from persons with relapse cases (*2*,*5*). This finding suggests that virus might persist in a noninfectious mutant form analogous to that which occurs with related measles virus during subacute sclerosing panencephalitis (*14*). In addition, the cellular reservoir for the virus and the mechanism by which it maintains latency are unknown.

It would be desirable to use PCR to test a variety of sample types for diagnosis of infection with Hendra virus. All sample types tested were suitable in terms of sensitivity. However, obtaining an NPA specimen during the acute phase of infection has a potential risk for aerosol contamination, and needle stick injury is a risk if a blood sample is obtained. Collection of a urine sample may be an acceptable risk without reducing the sensitivity of detection and risk to health care staff. This procedure should assist clinicians in management of henipavirus infections.
